# Impact of Medical Comorbidities on Respiratory-Related Patient-Reported Outcome Measures in Fibrotic Interstitial Lung Disease

**DOI:** 10.3390/jcm15031051

**Published:** 2026-01-28

**Authors:** Joon Yong Moon, Madison Beenken, Teng Moua

**Affiliations:** 1 Division of Pulmonary and Critical Care Medicine, Mayo Clinic, Rochester, MN 55905, USA; moon.joonyong@mayo.edu; 2 Division of Biostatistics, Mayo Clinic, Rochester, MN 55905, USA; beenken.madison@mayo.edu

**Keywords:** fibrotic interstitial lung disease, medical comorbidities, patient-reported outcome measures, chronic respiratory questionnaire

## Abstract

**Background/Objectives:** Individual and increasing numbers of comorbidities have been associated with worse outcomes in patients with fibrotic interstitial lung disease (f-ILD). The association and impact of medical comorbidities on patient-reported outcome measures (PROMs) in f-ILD have yet to be reported. **Methods:** Analysis was conducted using data from a single-center prospective cohort involving 199 patients with f-ILD. All f-ILD diagnoses and severities were screened and enrolled over a three-year study period. Baseline demographics, pulmonary function test (PFT) measures, and survival status were collected. PROMs, including the Chronic Respiratory Questionnaire (CRQ) and the Self-Management Ability Scale (SMAS-30), were assessed at baseline and serially. Thirteen medical comorbidities were evaluated for their prevalence and impact on PROMs and all-cause mortality. **Results:** Mean age was 69 years, with a female-to-male ratio of 61% vs. 39%. Dyslipidemia (74%) and gastroesophageal reflux disease (GERD) (71%) were the most prevalent comorbidities. Hypertension, diabetes, GERD, pulmonary hypertension (PH), depression, congestive heart failure (CHF), and obstructive sleep apnea (OSA), were independently associated with lower PROM scores along with increasing numbers of concomitant comorbidities. Increasing numbers of comorbidities, as well as specifically diabetes, PH, hypertension, CHF, and OSA, were associated with greater all-cause mortality. **Conclusions:** Medical comorbidities may independently impact respiratory-related PROMs in patients with f-ILD. These findings highlight the importance of comprehensive comorbidity management in improving quality of life and survival outcomes in patients with f-ILD.

## 1. Introduction

The fibrotic interstitial lung diseases (f-ILDs) are characterized by recurrent lung injury with fibrosis and progressive shortness of breath, cough, and loss of quality of life [1,2]. They are heterogeneous in terms of pathologic mechanisms and presentation with current medical treatments only slowing or stabilizing loss of lung function. Few treatments have directly impacted presenting symptoms or loss of respiratory-related quality of life [1,3,4].

Traditional measures of disease progression or severity in f-ILD have included worsening symptoms, decline in pulmonary function testing (PFT), and increased radiologic abnormalities. Unfortunately, correlation of objective measures (PFT and computed tomography (CT) findings) with clinical symptoms or disease burden may be difficult when the disease is mid-range and neither early nor advanced [5,6,7]. In mid-range fibrosis, compared to the extremes of the disease, heterogeneous and regionally variable lung involvement produces non-linear physiologic and radiologic changes that are not well captured by objective measures, such as forced vital capacity (FVC) [8]. This limitation strengthens the rationale for multidimensional assessment, incorporating symptoms, physiology, and imaging together.

Patient-reported outcome measures (PROMs) are self-reported surveys or questionnaires that directly assess symptom burden and/or health-related quality of life without the interpretation or involvement of an intermediary [9]. They may provide a better understanding of specific health-related quality of life domains and insights into the impact of related and unrelated medical comorbidities [10,11].

There are no prior assessments of the direct impact or association of medical comorbidities on respiratory-related PROMs in patients with f-ILD. Little data also exists on the impact of medical comorbidities on PROMs in patients with other chronic lung diseases. Prior studies in patients with chronic obstructive pulmonary disease (COPD) have suggested that increasing numbers of comorbidities or specific comorbidities (such as congestive heart failure or depression) may directly impact PROMs [12,13,14]. In IPF, cardiac disease and heart failure occur more frequently than in matched COPD cohorts, and patients with IPF live, on average, only two-thirds as long as those with COPD after diagnosis, suggesting distinct prognostic profiles in those with fibrosis [15]. We hypothesize that medical comorbidities will similarly impact PROMs in patients with f-ILD and contribute to increased all-cause mortality, though collinearity with other predictors such as age or lung function may be relevant. In this study, we used the Chronic Respiratory Questionnaire (CRQ) and the Self-Management Ability Scale (SMAS-30) for assessing the impact of comorbidities on PROMs and all-cause mortality in patients with f-ILD.

## 2. Materials and Methods

### 2.1. Study Design, Setting, and Participants

The current study is a post hoc analysis of a previously reported prospective cohort assessing baseline and serially measured PROMs in patients with f-ILD [16]. Institutional review board (IRB) approval was obtained prior to study initiation (Mayo IRB 17-005475). Written informed consent was obtained from all participants, and data were stored securely and handled in accordance with institutional policies for patient privacy and confidentiality. Study patients were enrolled from 2018 to 2021 and included 199 patients followed over the study period through October 2023. Patients with f-ILD, defined by greater than 10% fibrosis on chest CT as assessed by radiology and clinical colleagues, were enrolled, with no exclusions made for disease subtype, PFT severity, or treatment. Collated demographics included age at study enrollment, sex, smoking history, and f-ILD subtype (idiopathic pulmonary fibrosis (IPF) and all non-IPF). PFT measures were baseline percent predicted forced vital capacity (FVC%) and diffusion capacity for carbon monoxide (DLCO%) closest to the date of study enrollment and at the time of subsequent protocolized PROM assessments (within one month or 30 days before or after). Additional survival status after the three-year observation period was collected from the electronic medical record, and if lost to follow-up, confirmed with a search of the United States Social Security Index (last search date 22 October 2024). Patients lost to follow-up or voluntarily withdrew were not included in the analysis, with enrolled patients requiring 2 or more PROM and PFT measures for linear regression models.

### 2.2. Patient-Reported Outcome Measures

PROMs were administered by send-out paper format or in-person and collected at the time of study enrollment (baseline), 3, 6, and 12 months in patients with IPF and non-IPF disease. The Chronic Respiratory Questionnaire (CRQ) is a validated 20-item questionnaire assessing four domains of respiratory-related quality of life in patients with chronic lung disease. These domains include Dyspnea, Fatigue, Emotion, and Mastery. Individual items are scored via a seven-point Likert scale, with higher scores representing less impairment or greater functionality [17,18]. Individual CRQ domains were assessed, as well as the Dyspnea and Fatigue domains combined for a Physical summary score and the Emotion and Mastery domains combined for an Emotional Summary score, as previously performed in other studies for assessing dual summary domain effects for the CRQ in f-ILD and other chronic lung disease [16,19,20,21].

The Self-Management Ability Scale (SMAS-30) is a 30-item questionnaire used to assess participant self-management ability, highlighting health behaviors such as self-efficacy, investment behavior, and taking initiative [22]. A total score on a 100-point scale is calculated as the average of the six subscales, with higher scores suggesting better self-management ability. SMAS-30 has been correlated with loss of general function often associated with increased risk of hospitalization, while subscales of ‘taking initiative’, ‘investment behavior’, ‘self-efficacy’, ‘variety’, and ‘multifunctionality’ have been correlated with various aspects of physical and social well-being [23]. Disease severity or progression with either gain or loss in the ability to cope or manage such changes has been assessed with SMAS-30 [24].

### 2.3. Comorbidity Selection and Definitions

A systematic review of the published literature was performed to assess the type and prevalence of relevant medical comorbidities associated with f-ILD. Prior et al. reviewed ILD registries from Denmark and Germany collected between 2003 and 2018, and highlighted the prevalence and impact of 20 comorbidities [25]. Among these, a population prevalence of more than 5% was used for potential inclusion in our study. After adjudication by the study team, thirteen medical comorbidities were selected for analysis: (1) hypertension (HTN), (2) diabetes, (3) gastroesophageal reflux disease (GERD), (4) coronary or cardiovascular disease (CAD or CVD), (5) dyslipidemia, (6) atrial fibrillation (A-fib), (7) osteoporosis, (8) pulmonary hypertension (PH), (9) depression, (10) congestive heart failure (CHF), (11) cancer (all stages of solid and non-solid organ, excluding non-melanotic skin cancers), (12) thyroid disease, and (13) obstructive sleep apnea (OSA). Individual patient records were reviewed for predefined comorbidities and included based on 2 of 3 criteria: listed problem list or past medical history, ICD-10 code, or documentation of disease-specific therapy. While the comparative clinical or long-term impact of individual comorbidities was also considered (for example, CHF may portend greater clinical impact and morbidity than osteoporosis or thyroid disease), given the absence of comorbidity-specific quality-of-life measures for selected comorbidities and varying comorbidity stages or severities, weighing or ranking individual comorbidities according to their potential impact on PROMs was not pursued. Comorbidities were also only included if they were diagnosed prior to or during the study period and associated with the timing of a PROM measure. Newly diagnosed comorbidities occurring after the 3-year observation period were excluded from the analysis of PROM impact but included in the assessment of all-cause mortality outcomes.

### 2.4. Statistical Methods

Continuous data were presented as mean ± standard deviation (SD), while categorical data were presented as frequency and percentage. Baseline comparisons between patients with IPF and non-IPF were made using two sample *t*-tests for continuous variables and chi-square tests for categorical variables. Linear regression models were used to assess the effect of an individual comorbidity or the number of comorbidity diagnoses on PROM scores at baseline. These results are presented as point estimates with 95% confidence intervals. Adjustments were made for a priori covariables of age (at study enrollment or comorbidity diagnosis if occurring within the 3-year observation period), percent predicted FVC and DLCO, and f-ILD subtype (IPF vs. non-IPF) on multivariable analysis. Univariable and multivariable Cox regression were used to assess the association of individual comorbidities as well as the total number of comorbidity diagnoses on all-cause mortality over time, adjusted for the same a priori covariables. An exploratory analysis was performed using Kaplan–Meier survival curves to compare survival rates for patients based on the number of concomitant comorbidities by quartiles. These groups were compared using pairwise log-rank tests with a Holm–Bonferroni correction for multiple testing. *p* values less than 0.05 were considered statistically significant and all statistical analysis was completed using R (version 4.5.3; R Core Team (2025)).

## 3. Results

### 3.1. Patient Characteristics

Of 304 patients screened, 199 were enrolled. Mean age was 69 ± 9, with a higher female-to-male ratio (61% vs. 39%) (Table 1). A third of patients were diagnosed with IPF (33%), with fibrotic hypersensitivity pneumonitis and connective-tissue disease-related ILD comprising 21% and 18% of the cohort, respectively. Slightly more than half were active or former smokers (55%). Forty-six percent of IPF and 63% of non-IPF f-ILD patients completed all protocolized interval PROMs over the one-year measurement period, with 77% of the cohort completing 6-month interval measures. All-cause mortality for the study period was 44%. Prevalence of individual comorbidities ranged from 18% to 74%, with dyslipidemia (74%) and GERD (71%) being the two most frequent medical comorbidities for the whole cohort.

### 3.2. Impact of f-ILD Subtype on PROMs

Two CRQ domain scores were higher in patients with IPF than non-IPF (CRQ Dyspnea (5.33 vs. 4.84, *p* = 0.041), Emotion (5.27 vs. 4.87, *p* = 0.025) suggesting better self-reported respiratory-related quality of life. Higher Physical (4.9 vs. 4.46, *p* = 0.027), and Emotional Summary (5.29 vs. 4.92, *p* = 0.03) scores were also found in patients with IPF. Total and individual SMAS-30 domain scores were similar between the two groups (Table 2).

### 3.3. Impact of Individual Comorbidities on PROM Domains and Scores

Multivariable linear regression assessing the number and impact of individual comorbidities on selected PROM domain and total scores is presented in Table 3. Concomitant adjustments were made for a priori covariables of age, sex, FVC%, and IPF diagnosis. Increasing numbers of concomitant comorbidities were associated with lower scores for all PROM domains and subscores except the SMAS-30 ‘positive frame of mind.’ Several individual comorbidities were independently associated with lower CRQ and SMAS-30 domain scores. Hypertension was associated with lower CRQ Dyspnea domain scores (−0.43, *p* = 0.041) while diabetes was associated with lower CRQ Dyspnea (−0.47, *p* = 0.033), CRQ Fatigue (−0.39, *p* = 0.042), CRQ Physical Summary (−0.42, *p* = 0.026), SMAS-30 Taking Initiative (−5.07, *p* = 0.036), SMAS-30 Behavior (−6.52, *p* = 0.013), SMAS-30 Self-Efficacy (−4.24, *p* = 0.027), and SMAS-30 Total (−4.48, *p* = 0.015) scores. GERD was associated with lower CRQ Fatigue scores (−0.42, *p* = 0.044). Patients with PH reported lower CRQ Dyspnea (−0.50, *p* = 0.019) and CRQ Fatigue (−0.50, *p* = 0.019) scores. Depression was also associated with lower CRQ Fatigue (−0.51, *p* = 0.011), CRQ Emotional Summary (−0.53, *p* = 0.002), SMAS-30 Taking Initiative (−6.99, *p* = 0.005), SMAS-30 Behavior (−7.09, *p* = 0.009), and SMAS-30 Self-Efficacy (−5.42, *p* = 0.006), and SMAS-30 Total (−5.71, *p* = 0.003) scores. CHF was notably associated with lower scores across all PROM domains and subscores. Lastly, individuals with OSA reported lower CRQ Dyspnea (−0.75, *p* = 0.001), CRQ Fatigue (−0.57, *p* = 0.0017), CRQ Physical Summary (−0.68, *p* = 0.001), CRQ Emotional Summary (−0.44, *p* = 0.004), SMAS-30 Behavior (−6.75, *p* = 0.007), and SMAS-30 Self-Efficacy (−5.52, *p* = 0.002) scores. Notably, cancer appeared to be associated with a positive impact on CRQ Dyspnea (0.68, *p* = 0.016) and CRQ Physical Summary (0.48, *p* = 0.045) scores.

### 3.4. Impact of Comorbidities on All-Cause Mortality

Cox proportional hazards regression was used to assess the association of individual comorbidities with all-cause mortality, unadjusted and adjusted for similar a priori covariables of age, sex, FVC%, and IPF diagnosis, as presented in Table 4. An increasing number of concomitant comorbidities (HR 1.15 (1.05–1.26), *p* = 0.002), diabetes (HR 1.63 (1.05–2.54), *p* = 0.028), PH (HR 1.63 (1.05–2.52), *p* = 0.030), CHF (HR 1.80 (1.10–2.95), *p* = 0.020), and OSA (HR 1.80 (1.15–2.83), *p* = 0.010) were independently associated with increased mortality risk.

**Figure 1:**
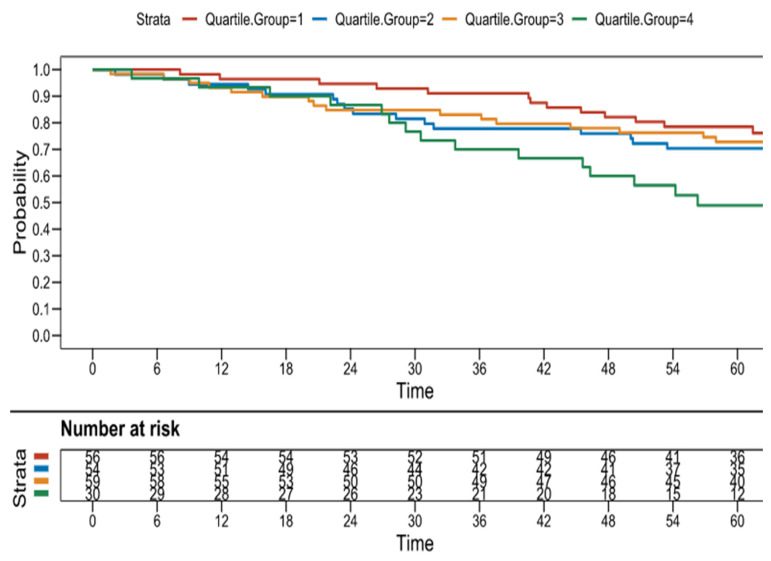
Kaplan–Meier survival curves according to the number of comorbidities (stratified by quartiles). Log-rank *p* = 0.001 (quartile 4 vs. quartile 1); Log rank *p* = 0.013 (quartile 2 vs. 4). [red = quartile 1 (0–3 comorbidities); blue = quartile 2 (4–5 comorbidities); orange = quartile 3 (6–7 comorbidities); green = quartile 4 (≥8 comorbidities)].

Kaplan–Meier curves for an increasing number of concomitant comorbidities as stratified by quartile frequency, are presented in Figure 1. Survival was better for those in quartiles 1 and 2 vs. quartile 4 (*p* = 0.001 and 0.013, respectively), highlighting worse survival for quartile 4 in patients with the greatest number of concomitant medical comorbidities (8 to 12).

## 4. Discussion

To the best of our knowledge, this study highlights for the first time the impact of specific medical comorbidities on PROMs in patients with f-ILD. Our findings also confirm the known association of specific and increasing numbers of comorbidities with all-cause mortality. We found that comorbidities, including depression, HTN, diabetes, GERD, PH, CHF, and OSA, were negatively associated with selected respiratory and non-respiratory-related PROMs, independent of underlying lung disease severity or other predictors of disease progression. Conditions such as diabetes, PH, HTN, CHF, and OSA were also associated with increased all-cause mortality. These findings extend prior work that comorbidities may add to or exacerbate disease burden in f-ILD and lead to worse health outcomes [7,26]. The importance of addressing comorbid conditions as part of comprehensive management in patients with f-ILD remains relevant as lung-specific therapies are limited and do not currently reverse fibrosis.

PROMs and comorbidities have been associated with disease severity [16] and long-term outcomes [27,28] in patients with f-ILD. The impact of comorbidities on PROMs themselves, though, has not been previously reported. In other chronic lung diseases such as COPD, concomitant depression has been associated with poorer health-related quality of life and increased symptom burden, as assessed by the Hospital Anxiety and Depression Scale and St. George’s Respiratory Questionnaire (SGRQ) [12]. One study found that comorbidities such as CHF, hyperlipidemia, and depression were associated with higher SGRQ scores (greater respiratory-related disease burden), more frequent exacerbations, and increased mortality in patients with mild-to-moderate COPD [29]. Similarly, in patients with end-stage renal disease, those with multiple comorbidities reported decreased HR-QoL, particularly in physical health domains [30]. These results parallel the potential impact of comorbidities on PROMs in f-ILD. For example, diabetes, CHF, OSA, and depression show negative effects across nearly all PROM domains. In contrast, a positive association between cancer and CRQ Dyspnea and Physical Summary scores was not expected. This finding may reflect symptom reframing among patients with treated or stable malignancy or a healthy-survivor effect; however, careful interpretation is advised as our cohort included diverse cancer types and disease stages, which likely vary in terms of symptom burden and disease trajectory.

Our findings are consistent with the prior literature, reporting the negative impact of comorbidities on mortality in patients with f-ILD [26,31]. Recent systematic reviews in IPF have identified PH, OSA, HTN, and CHF as contributing to greater mortality, along with lung cancer, pulmonary embolism, CAD, and GERD [27,32]. We reviewed the impact of comorbidities on outcomes in both IPF and non-IPF f-ILD and found similar trends [27,32,33]. A study reviewing the impact of comorbidities in f-ILD described greater numbers of comorbidities associated with increased mortality but did not report the specific impact of individual comorbidities [31]. HTN, diabetes, CAD, lung cancer, and COPD were more prevalent in the deceased group. Kreuter et al. reported a median survival of 66 months in patients with IPF with no comorbidities, 48 months in those with one to three comorbidities, and 35 months in those with four or more comorbidities [27].

PROMs remain of relevance in clinical practice as well as research, where they often serve as secondary endpoints in clinical trials [6,9,11]. Despite their widespread use, clinical trials in f-ILD have reported little to no impact on PROMs, even when medical therapies may have demonstrated a positive impact on reducing lung function decline [3,34]. This is likely due to the varying severity of already present disease burden at the time of study enrollment and its likely irreversibility with treatments targeting primarily lung function stabilization. In future studies where statistical endpoints are met for efficacy related to lung function, secondary PROM endpoints may be confounded by the presence and number of comorbidities, limiting accurate assessments of patient-centered efficacy. Our study offers a quantitative assessment of this association involving a wide spectrum of comorbidities but a limited selection of PROMs. Accounting for PROM scores and comorbid conditions in future interventional studies may vary based on which comorbidities and PROMs are being assessed.

Our study has several limitations. As it was conducted at a single-center at a tertiary referral center, generalizability may be limited, particularly to community or other practice settings. In addition, treatment exposures may confound PROM interpretation, as antifibrotic and immunosuppressive therapies often contribute their own adverse effects, such as increased fatigue or gastrointestinal symptoms, impacting PROM scores. Clinical severity and duration of selected comorbidities may be difficult to assess in terms of their dimensional impact on respiratory-related or self-management outcomes. Disease-specific severity or impact scores were not available for all comorbidities and may not be comparable or scalable between diseases. Quality of life, for example, as reported by patients with stage IV metastatic cancer, may differ significantly from that of those with well-controlled HTN, even if respiratory-related symptom burden is the same. This variation may depend on the severity or staging of cancer at presentation, with the additional difficulty of comparing cancer to non-cancer comorbidities or the number of combined comorbidities [27,32]. Nonetheless, associations were noted for specific comorbidities and specific domains on PROM testing, even after adjusting for underlying lung disease. f-ILD severity may also be related to older age or longer disease duration, on its own, increasing the likelihood of comorbidity development. We accounted for this with adjustments for age, baseline FVC%, and disease subtype (IPF vs. non-IPF), using a large prospective cohort representing several geographic regions from a single tertiary institution. Lastly, while we looked specifically at CRQ and SMAS-30 as specific PROMs for our f-ILD cohort, comorbidity impact on other PROMs warrants separate evaluations to determine item-specific extent and direction of any associations.

## 5. Conclusions

In summary, we highlight the individual and collective impact of comorbidities on respiratory-related quality of life and self-management PROMs in patients with f-ILD. Specific comorbidities such as HTN, diabetes, PH, CHF, and OSA were associated with lower or more negative respiratory-related quality of life and self-management ability, while diabetes, PH, CHF, and OSA were also associated with greater all-cause mortality. Our findings suggest comprehensive approaches incorporating treatment strategies that address related and unrelated comorbidities may not only impact survival but also the use of PROMs as targets of therapy, in clinical practice and research.

## Figures and Tables

**Table 1:** Cohort characteristics and comorbidities.

Characteristic	Finding
Age (years), mean ± SD (range)	69 ± 9 (32–88)
Sex, M/F (%/%)	78/121 (39/61)
FVC%, mean ± SD (range)	71 ± 20 (19–133)
DLCO%, mean ± SD (range)	49 ± 17 (19–102)
Smoker status, N (%) Active Former Never	6 (3) 104 (52) 89 (45)
f-ILD subtype, N (%) Idiopathic pulmonary fibrosis Fibrotic hypersensitivity pneumonitis Connective-tissue disease-associated ILD Other idiopathic interstitial pneumonia Combined pulmonary fibrosis and emphysema Occupational/Drug-induced Fibrotic sarcoid Unclassifiable/atypical	65 (33) 41 (21) 35 (18) 6 (3) 9 (5) 4 (2) 1 (1) 38 (19)
Mortality, N (%)	87 (44)
Comorbidities, N (%) Hypertension Diabetes GERD CAD or CVD Dyslipidemia Atrial fibrillation Osteoporosis Pulmonary Hypertension CHF Depression Cancer (all) OSA Thyroid disease	111 (56) 65 (33) 143 (71) 88 (44) 147 (74) 38 (19) 40 (20) 93 (47) 41 (21) 41 (21) 36 (18) 96 (48) 45 (23)

Abbreviations: FVC = forced vital capacity, DLCO = diffusion capacity for carbon monoxide, f-ILD = fibrotic interstitial lung disease, GERD = gastroesophageal reflux disease, CAD = coronary artery disease, CVD = cardiovascular disease, CHF = congestive heart failure, OSA = obstructive sleep apnea.

**Table 2:** Baseline PROMs by f-ILD Subtype (IPF vs. non-IPF).

PROM, Mean ± Standard Deviation (Range)	IPF (N = 65)	Non-IPF (N = 134)	*p*-Value
CRQ Dyspnea	5.33 ± 1.51 (1.8–7)	4.84 ± 1.59 (1–7)	0.041
CRQ Fatigue	4.37 ± 1.24 (1.3–6.8)	3.99 ± 1.3 (1–7)	0.061
CRQ Emotion	5.27 ± 1.19 (1.1–7)	4.87 ± 1.1 (1.9–6.9)	0.025
CRQ Mastery	5.36 ± 1.28 (1–7)	4.99 ± 1.32 (1.3–7)	0.068
CRQ Physical Summary	4.9 ± 1.26 (1.6–6.8)	4.46 ± 1.34 (1.2–7)	0.027
CRQ Emotional Summary	5.29 ± 1.15 (1.1–7)	4.92 ± 1.11 (1.6–6.9)	0.030
SMAS-30 Taking initiative	72 ± 16 (40–100)	70 ± 16 (28–100)	0.414
SMAS-30 Behavior	66 ± 18 (24–100)	65 ± 17 (28–100)	0.61
SMAS-30 Variety	59 ± 13 (36–92)	59 ± 14 (20–96)	0.885
SMAS-30 Multifunctionality	77 ± 11 (48–100)	75 ± 11 (36–100)	0.335
SMAS-30 Self-efficacy	89 ± 12 (40–100)	88 ± 19 (36–100)	0.508
SMAS-30 Positive frame of mind	71 ± 19 (32–100)	69 ± 19 (36–100)	0.59
SMAS-30 Total	72 ± 12 (48–96)	71 ± 12 (36–95)	0.501

Abbreviations: PROM = patient-reported outcome measure, f-ILD = fibrotic interstitial lung disease, IPF = idiopathic pulmonary fibrosis, CRQ = Chronic Respiratory Questionnaire, SMAS-30 = Self-Management Assessment Scale-30.

**Table 3:** Multivariable linear regression model assessing the association of individual comorbidities on PROM scores.

Adjusted, Estimate (95% CI)	CRQ Dyspnea	CRQ Fatigue	CRQ Physical Summary	CRQ Emotional Summary	SMAS-30 Taking Initiative	SMAS-30 Behavior	SMAS-30 Self-Efficacy	SMAS-30 Total
Number of comorbidities	−0.17 (−0.25, −0.08) *	−0.12 (−0.2, 0.04) *	−0.14 (−0.22, 0.07) *	−0.08 (−0.15, −0.02) *	−1.01 (−1.99, 0.03) *	−1.39 (−2.46, −0.32) *	−1.05 (−1.81, −0.28) *	−0.92 (−1.66, −0.17) *
Hypertension	−0.43 (−0.85, −0.02) *	−0.16 (−0.53, 0.21)	−0.30 (−0.66, 0.05)	−0.17 (−0.47, 0.14)	0.52 (−4.05, 5.1)	−1.45 (−6.49, 3.59)	−2.57 (−6.17, 1.02)	−1.19 (−4.68, 2.29)
Diabetes	−0.47 (−0.91, −0.04) *	−0.39 (−0.78, −0.01) *	−0.42 (−0.79, 0.05) *	−0.25 (−0.57, 0.08)	−5.07 (−9.82, −0.32) *	−6.52 (−11.7, −1.38) *	−4.24 (−7.99, −0.49) *	−4.48 (−8.09, −0.88) *
GERD	−0.32 (−0.79, 0.13)	−0.42 (−0.82, −0.01) *	−0.35 (−0.74, 0.05)	−0.32 (−0.66, 0.03) 9	−3.31 (−8.34, 1.71)	−1.21 (−6.77, 4.36)	−0.38 (−4.37, 3.61)	−2.06 (−5.90, 1.78)
CAD or CVD	−0.25 (−0.68, 0.18)	0.04 (−0.34, 0.42)	−0.10 (−0.47, 0.27)	0.01 (−0.32, 0.33),	−2.74 (−7.43, 1.94)	−1.26 (−6.45, 3.92)	−2.29 (−5.99, 1.41)	−1.77 (−5.35, 1.82)
Dyslipidemia	−0.11 (−0.58, 0.37)	−0.01 (−0.43, 0.41)	−0.07 (−0.49, 0.32)	−0.01 (−0.36, 0.34)	−1.86 (−7.03, 3.32)	−0.61 (−6.33, 5.11)	0.60 (−3.49, 4.69)	−0.94 (−4.89, 3.01)
Atrial fibrillation	−0.28 (−0.82, 0.25),	−0.29 (−0.77, 0.18)	−0.29 (−0.75, 0.17)	−0.08 (−0.48, 0.32)	−0.09 (−5.98, 5.81)	−2.48 (−8.98, 4.01)	−1.32 (−5.97, 3.34)	−0.80 (−5.30, 3.69)
Osteoporosis	0.155 (−0.39, 0.69)	0.15 (−0.39, 0.69)	0.005 (−0.46, 0.47),	−0.02 (−0.43, 0.38)	0.64 (−5.29, 6.57)	−2.71 (−9.25, 3.82)	1.80 (−2.88, 6.48)	−0.50 (−5.03, 4.03)
Pulmonary hypertension	−0.50 (−0.92, −0.08) *	−0.50 (−0.92, −0.08) *	−0.30 (−0.66, 0.06)	−0.01 (−0.33, 0.31)	0.68 (−3.96, 5.33)	−0.96 (−6.09, 4.16)	0.65 (−3.02, 4.32)	0.678 (−2.87, 4.22)
Depression	−0.25 (−0.70, 0.19)	−0.51 (−0.90, −0.12) *	−0.37 (−0.76, 0.009)	−0.53 (−0.85, −0.19) *	−6.99 (−11.8, −2.16) *	−7.09 (−12.5, −1.74) *	−5.42 (−9.25, −1.59) *	−5.71 (−9.39, −2.03) *
CHF	−1.12 (−1.62, −0.62) *	−0.78 (−1.24, −0.33) *	−0.94 (−1.38, −0.51) *	−0.79 (−1.17, −0.42) *	−6.42 (−12.1, −0.71) *	−9.59 (−15.8, −3.37) *	−8.01 (−12.4, −3.59) *	−7.24 (−11.52, −2.95) *
Cancer	0.68 (0.13, 1.21) *	0.22 (−0.26, 0.71)	0.48 (0.01, 0.95) *	0.19 (−0.21, 0.59)	1.52 (−4.46, 7.50)	1.82 (−4.78, 8.43)	1.54 (−3.19, 6.27)	1.99 (−2.57, 6.56)
Thyroid	−0.28 (−0.77, 0.21)	−0.04 (−0.47, 0.39)	−0.17 (−0.59, 0.25)	0.16 (−0.19, 0.53)	0.143 (−5.2, 5.52)	3.00 (−2.92, 8.92)	−0.64 (−4.89, 3.61)	2.13 (−1.96, 6.23)
OSA	−0.75 (−1.15, −0.35) *	−0.57 (−0.93, −0.22) *	−0.68 (−1.03, −0.34) *	−0.44 (−0.74, −0.14) *	−2.70 (−7.22, 1.82)	−6.75 (−11.7, −1.83) *	−5.52 (−9.01, −2.02 *	−3.31 (−6.74, 0.12)

Adjusted for a priori covariables of age, sex, FVC%, and IPF vs. non-IPF diagnosis; * *p* value < 0.05, Abbreviations: CRQ = Chronic Respiratory Questionnaire, SMAS-30 = Self-Management Assessment Scale-30, PROM = patient-reported outcome measure, GERD = gastroesophageal reflux disease, CAD = coronary artery disease, CVD = cardiovascular disease, CHF = congestive heart failure, OSA = obstructive sleep apnea.

**Table 4:** Cox regression model assessing the association of comorbidities with all-cause mortality.

Comorbidity	Univariable, HR (95% CI)	*p*-Value	Multivariable, HR (95% CI)	*p*-Value
Number of comorbidities	1.17 (1.07, 1.27)	<0.001	1.15 (1.05, 1.26)	0.002
Hypertension	1.53 (0.99, 2.35)	0.056	1.47 (0.95, 2.27)	0.083
Diabetes	1.58 (1.03, 2.43)	0.038	1.63 (1.05, 2.54)	0.028
GERD	1.16 (0.72, 1.86)	0.500	1.18 (0.73, 1.92)	0.500
CAD or CVD	1.48 (0.97, 2.25)	0.069	1.26 (0.81, 1.95)	0.300
Dyslipidemia	1.99 (1.14, 3.47)	0.015	1.66 (0.95, 2.90)	0.076
Atrial Fibrillation	1.86 (1.15, 3.00)	0.011	1.27 (0.77, 2.10)	0.400
Osteoporosis	1.30 (0.80, 2.13)	0.300	1.19 (0.70, 2.03)	0.500
Pulmonary Hypertension	1.93 (1.26, 2.96)	0.003	1.63 (1.05, 2.52)	0.030
Depression	1.28 (0.83, 1.98)	0.300	1.59 (1.00, 2.51)	0.050
CHF	1.84 (1.15, 2.93)	0.010	1.80 (1.10, 2.95)	0.020
Cancer	0.70 (0.39, 1.26)	0.200	0.56 (0.31, 1.03)	0.064
Thyroid disease	0.78 (0.46, 1.32)	0.300	0.98 (0.57, 1.69)	>0.90
OSA	1.38 (0.90, 2.10)	0.140	1.80 (1.15, 2.83)	0.010

Adjusted for a priori covariables of age, sex, FVC%, and IPF vs. non-IPF diagnosis. HR = hazard ratio, GERD = gastroesophageal reflux disease, CAD = coronary artery disease, CVD = cardiovascular disease, CHF = congestive heart failure, OSA = obstructive sleep apnea.

## Data Availability

The data that support the findings of this study are available from the corresponding author upon reasonable request.

## Abbreviations

A-fib	atrial fibrillation
CAD	coronary artery disease
CHF	congestive heart failure
CPFE	combined pulmonary fibrosis and emphysema
CRQ	Chronic Respiratory Questionnaire
COPD	chronic obstructive pulmonary disease
CT	computed tomography
CTD-ILD	connective tissue disease-associated interstitial lung disease
CVD	cardiovascular disease
DLCO	diffusion capacity for carbon monoxide
f-HP	fibrotic hypersensitivity pneumonitis
f-ILD	fibrotic interstitial lung disease
FVC	forced vital capacity
GERD	gastroesophageal reflux disease
HR	hazard ratio
HR-QoL	health-related quality of life
HTN	hypertension
IIP	idiopathic interstitial pneumonia
IPF	idiopathic pulmonary fibrosis
OSA	obstructive sleep apnea
PFT	pulmonary function testing
PH	pulmonary hypertension
PROM	patient-reported outcome measure
SMAS-30	Self-Management Assessment Survey 30
TLC	total lung capacity

## References

[1] Raghu G., Remy-Jardin M., Myers J.L., Richeldi L., Ryerson C.J., Lederer D.J., Behr J., Cottin V., Danoff S.K., Morell F. (2018). Diagnosis of Idiopathic Pulmonary Fibrosis An Official ATS/ERS/JRS/ALAT Clinical Practice Guideline. Am. J. Respir. Crit. Care Med..

[2] Raghu G., Remy-Jardin M., Richeldi L., Thomson C.C., Inoue Y., Johkoh T., Kreuter M., Lynch D.A., Maher T.M., Martinez F.J. (2022). Idiopathic Pulmonary Fibrosis (an Update) and Progressive Pulmonary Fibrosis in Adults: An Official ATS/ERS/JRS/ALAT Clinical Practice Guideline. Am. J. Respir. Crit. Care Med..

[3] Flaherty K.R., Wells A.U., Cottin V., Devaraj A., Walsh S.L.F., Inoue Y., Richeldi L., Kolb M., Tetzlaff K., Stowasser S. (2019). Nintedanib in Progressive Fibrosing Interstitial Lung Diseases. N. Engl. J. Med..

[4] King T.E., Bradford W.Z., Castro-Bernardini S., Fagan E.A., Glaspole I., Glassberg M.K., Gorina E., Hopkins P.M., Kardatzke D., Lancaster L. (2014). A phase 3 trial of pirfenidone in patients with idiopathic pulmonary fibrosis. N. Engl. J. Med..

[5] Swigris J.J., Brown K.K., Abdulqawi R., Buch K., Dilling D.F., Koschel D., Thavarajah K., Tomic R., Inoue Y. (2018). Patients’ perceptions and patient-reported outcomes in progressive-fibrosing interstitial lung diseases. Eur. Respir. Rev..

[6] Kalluri M., Luppi F., Vancheri A., Vancheri C., Balestro E., Varone F., Mogulkoc N., Cacopardo G., Bargagli E., Renzoni E. (2021). Patient-reported outcomes and patient-reported outcome measures in interstitial lung disease: Where to go from here?. Eur. Respir. Rev..

[7] Kreuter M., Swigris J., Pittrow D., Geier S., Klotsche J., Prasse A., Wirtz H., Koschel D., Andreas S., Claussen M. (2017). Health related quality of life in patients with idiopathic pulmonary fibrosis in clinical practice: Insights-IPF registry. Respir. Res..

[8] Humphries S.M., Chung A., Swigris J.J., Oh A.S., Walsh S.L.F., Lynch D.A., Goldin J.G., Kim G.H.J. (2025). Quantification of Interstitial Lung Diseases, from the AJR Special Series on Quantitative Imaging. Am. J. Roentgenol..

[9] U.S. Department of Health and Human Services FDA Center for Drug Evaluation and Research, U.S. Department of Health and Human Services FDA Center for Biologics Evaluation and Research, U.S. Department of Health and Human Services FDA Center for Devices and Radiological Health (2006). Guidance for industry: Patient-reported outcome measures: Use in medical product development to support labeling claims: Draft guidance. Health Qual. Life Outcomes.

[10] Cox I.A., Borchers Arriagada N., de Graaff B., Corte T.J., Glaspole I., Lartey S., Walters E.H., Palmer A.J. (2020). Health-related quality of life of patients with idiopathic pulmonary fibrosis: A systematic review and meta-analysis. Eur. Respir. Rev..

[11] Aronson K.I., Danoff S.K., Russell A.M., Ryerson C.J., Suzuki A., Wijsenbeek M.S., Bajwah S., Bianchi P., Corte T.J., Lee J.S. (2021). Patient-centered Outcomes Research in Interstitial Lung Disease: An Official American Thoracic Society Research Statement. Am. J. Respir. Crit. Care Med..

[12] Burgel P.R., Escamilla R., Perez T., Carré P., Caillaud D., Chanez P., Pinet C., Jebrakh G., Brinchault G., Court-Fortune I. (2013). Impact of comorbidities on COPD-specific health-related quality of life. Respir. Med..

[13] Holle R., Wacker M.E., Joerres R.A., Schulz H., Heinrich J., Peters A., Koch A., Leidl R., Vogelmeier C.F., Grp C.S. (2015). Do Comorbidities Have A Specific Impact on Generic Health-Related Quality of Life (hrql) in COPD Patients Compared to Controls? First Results of The German Cosyconet Cohort Study. Am. J. Respir. Crit. Care Med..

[14] Park H.Y., Yoo K.H., Lee J.H., Kim D.K., Lee H., Lee J.D., Jung E.J., Jung K.S., Yeon J. (2017). Different Impact of Respiratory Symptoms and Comorbidities on Copd-Specific Health-Related Quality of Life by Copd Severity. Respirology.

[15] Ozaltin B., Chapman R., Arfeen M.Q.U., Fitzpatick N., Hemingway H., Direk K., Jacob J. (2024). Delineating excess comorbidities in idiopathic pulmonary fibrosis: An observational study. Respir. Res..

[16] Duke J.D., Roy M., Daley S., Hoult J., Benzo R., Moua T. (2024). Association of patient-reported outcome measures with lung function and mortality in fibrotic interstitial lung disease: A prospective cohort study. ERJ Open Res..

[17] Wijkstra P.J., TenVergert E.M., Van Altena R., Otten V., Postma D.S., Kraan J., Koeter G.H. (1994). Reliability and Validity of the Chronic Respiratory Questionnaire (Crq). Thorax.

[18] Schunemann H.J., Puhan M., Goldstein R., Jaeschke R., Guyatt G.H. (2005). Measurement properties and interpretability of the Chronic respiratory disease questionnaire (CRQ). J. Chronic Obstr. Pulm. Dis..

[19] Moua T., Kubbara A., Novotny P., Ridgeway J.L., Limper A.H., Ryu J.H., Clark M.M., Benzo R. (2021). Patient-reported quality of life in fibrotic interstitial lung disease: Novel assessments of self-management ability and affect. ERJ Open Res..

[20] Benzo R., Hoult J., McEvoy C., Clark M., Benzo M., Johnson M., Novotny P. (2022). Promoting Chronic Obstructive Pulmonary Disease Wellness through Remote Monitoring and Health Coaching: A Clinical Trial. Ann. Am. Thorac. Soc..

[21] Schunemann H.J., Guyatt G.H., Griffith L., Stubbing D., Goldstein R. (2002). A randomized controlled trial to evaluate the effect of informing patients about their pretreatment responses to two respiratory questionnaires. Chest.

[22] Schuurmans H., Steverink N., Frieswijk N., Buunk B.P., Slaets J.P., Lindenberg S. (2005). How to measure self-management abilities in older people by self-report. The development of the SMAS-30. Qual. Life Res..

[23] Cramm J.M., Strating M.M., de Vreede P.L., Steverink N., Nieboer A.P. (2012). Validation of the self-management ability scale (SMAS) and development and validation of a shorter scale (SMAS-S) among older patients shortly after hospitalisation. Health Qual. Life Outcomes.

[24] Lee J.Y.T., Tikellis G., Glaspole I., Khor Y.H., Symons K., Holland A.E. (2021). Self-management for pulmonary fibrosis: Insights from people living with the disease and healthcare professionals. Patient Educ. Couns..

[25] Prior T.S., Hyldgaard C., Torrisi S.E., Kronborg-White S., Ganter C., Bendstrup E., Kreuter M. (2022). Comorbidities in unclassifiable interstitial lung disease. Resp. Res..

[26] Alhamad E.H., Cal J.G., Alrajhi N.N., Aharbi W.M., AlRikabi A.C., AlBoukai A.A. (2020). Clinical characteristics, comorbidities, and outcomes in patients with idiopathic pulmonary fibrosis. Ann. Thorac. Med..

[27] Kreuter M., Ehlers-Tenenbaum S., Palmowski K., Bruhwyler J., Oltmanns U., Muley T., Heussel C.P., Warth A., Kolb M., Herth F.J.F. (2016). Impact of Comorbidities on Mortality in Patients with Idiopathic Pulmonary Fibrosis. PLoS ONE.

[28] Case A.H., Hellkamp A.S., Neely M.L., Bender S., Dilling D.F., Gulati M., Hotchkin D.L., Huie T.J., Lancaster L., Snyder L.D. (2020). Associations between Patient-reported Outcomes and Death or Lung Transplant in Idiopathic Pulmonary Fibrosis. Data from the Idiopathic Pulmonary Fibrosis Prospective Outcomes Registry. Ann. Am. Thorac. Soc..

[29] Lee H., Jhun B.W., Cho J., Yoo K.H., Lee J.H., Kim D.K., Lee J.D., Jung K.S., Lee J.Y., Park H.Y. (2017). Different impacts of respiratory symptoms and comorbidities on COPD-specific health-related quality of life by COPD severity. Int. J. Chronic Obstr. Pulm. Dis..

[30] Cha J., Han D. (2020). Health-Related Quality of Life Based on Comorbidities Among Patients with End-Stage Renal Disease. Osong Public Health Res. Perspect..

[31] Znegui T., Nadia M., Mahmoud N., Amina K., Nesrin K., Rahma G., Najla B., Walid F., Sameh M., Ilhem Y. (2021). Associations between comorbidities and survival in patients with interstitial lung diseases. Eur. Respir. J..

[32] Raghu G., Amatto V.C., Behr J., Stowasser S. (2015). Comorbidities in idiopathic pulmonary fibrosis patients: A systematic literature review. Eur. Respir. J..

[33] Torrisi S.E., Ley B., Kreuter M., Wijsenbeek M., Vittinghoff E., Collard H.R., Vancheri C. (2019). The added value of comorbidities in predicting survival in idiopathic pulmonary fibrosis: A multicentre observational study. Eur. Respir. J..

[34] Behr J., Prasse A., Kreuter M., Johow J., Rabe K.F., Bonella F., Bonnet R., Grohe C., Held M., Wilkens H. (2021). Pirfenidone in patients with progressive fibrotic interstitial lung diseases other than idiopathic pulmonary fibrosis (RELIEF): A double-blind, randomised, placebo-controlled, phase 2b trial. Lancet Respir. Med..

